# Hydrogen Gas Treatment Improves Postoperative Delirium and Cognitive Dysfunction in Elderly Noncardiac Patients

**DOI:** 10.3390/jpm13010067

**Published:** 2022-12-28

**Authors:** Hua Lin, Jian Du, Zhigang Tian, Yonghao Yu, Yan Cui, Keliang Xie

**Affiliations:** 1 Department of Anesthesiology, Tianjin Institute of Anesthesiology, Tianjin Medical University General Hospital, Tianjin 300052, China; 2 Department of Pathogen Biology, School of Basic Medical Sciences, Tianjin Medical University, Tianjin 300070, China; 3 Department of Critical Care Medicine, Tianjin Medical University General Hospital, Tianjin 300052, China

**Keywords:** pre-operative pain, postoperative delirium, C-reactive protein, cognitive dysfunction, the elderly

## Abstract

*Purpose*: Postoperative delirium is a state of acute brain dysfunction characterized by fluctuating mental status that affects millions of patients each year. We used prophylactic inhalation of hydrogen gas in elderly patients undergoing elective surgery to compare their occurrence of postoperative delirium with that of controls. *Methods*: A total of 184 patients aged ≥ 65 years were enrolled and randomized into either a control group or a hydrogen inhalation group. The quality of sleep was assessed 1 day before and 1, 3, and 7 days after surgery at 8 A.M. The Confusion Assessment Method (CAM) was used as a screening tool for delirium and assessed the patients’ state of consciousness 1–7 days after surgery. *Results*: Postoperative delirium occurred in 17 (24%) of 70 patients without hydrogen inhalation and in 10 (12%) of 83 patients after hydrogen inhalation. The incidence of delirium was decreased in the hydrogen group. No significant differences were found between length of stay in hospital after surgery and sleep quality at 1, 3, and 7 days postoperatively between the two groups. The numerical rating scale (NRS) pain scores were higher in the hydrogen group (4.08 ± 1.77) than the control group (3.54 ± 1.77) on day 1 (*p* < 0.05); however, the mean difference between the two groups was small (1 to 1.6). There were no significant differences on day 3 and 7. The postoperative C-reactive protein level was significantly lower in the hydrogen group than the control group. *Conclusions*: This study suggests that hydrogen inhalation can prevent postoperative delirium in elderly noncardiac patients by reducing the inflammatory response.

## 1. Introduction

Postoperative delirium (POD), a common complication after surgery in elderly individuals (aged 60 years and older), is a state of acute brain dysfunction characterized by fluctuating mental status that affects millions of patients each year. More than one-third of delirium cases occur in patients aged 65 years and older who are hospitalized for surgery [1]. Delirium is also associated with morbidity and mortality, increased hospital costs, and even further cognitive decline [2,3,4]. Postoperative outcomes have become a central concern, with postoperative delirium and cognitive decline leading not only to difficulty moving but also to an increased risk of falls and even post-traumatic stress disorder [4,5,6,7,8]. This also poses an enormous challenge for families [4]. Given the aging of the population in the coming years, the prevention and treatment of delirium is of paramount importance. There are currently three important aspects of delirium prevention: pre-operative screening for cognitive function, taking certain precautions against delirium, and avoiding peri-operative medications that aggravate delirium [1].

Delirium has been shown to be associated with neurological inflammation, and proinflammatory signaling molecules have been identified in both patients and animal models [9]. Bloodborne pro-inflammatory factors leading to a systemic inflammatory environment can have a severely negative impact on the central nervous system [10,11]. Monocytes, neutrophils, and other peripheral immune inflammatory factors in the circulatory system lead to changes in neuronal function, synaptic plasticity, and glial cell homeostasis associated with other immune inflammatory factors, yet the effects of such factors have not been investigated [9].

Hydrogen is a selective anti-oxidant that can be effective in the treatment of many diseases [12,13]. Our previous studies found that hydrogen prevents sepsis-induced brain damage [13,14], and in animal models, hydrogen was also found to alleviate sepsis-induced cognitive dysfunction [13,14,15]. At the same time, no significant side effects of hydrogen treatment were found by testing various physiological indicators in animals. Hydrogen is now available as a medical gas for clinical patients, and the latest development of a high concentration hydrogen inhalation device, the hydrogen–oxygen nebulizer, combines the advantages of conventional electrolysis and ion membrane technology by adding AC/DC conversion to the two electrodes of a pure water electrolysis device, which can output a gas mixture of 66.6% hydrogen and 33.3% oxygen at different flow rates for clinical selection. However, there are no studies on the prevention of and reduction in postoperative delirium in elderly patients undergoing surgery; consequently, we chose prophylactic inhalation of hydrogen gas in elderly patients undergoing elective surgery to compare the occurrence of postoperative delirium with that in controls.

## 2. Materials and Methods

### 2.1. Study Design

We conducted this pragmatic, randomized, double-blind, and controlled clinical trial at Tianjin Medical University General Hospital in Tianjin, China, from January 2021 to October 2021. The first case was recorded on 10 January 2021. The study was approved by the Medical Ethics Committee of the General Hospital of Tianjin Medical University (IRB2020-YX-061-01) and registered in the Chinese Clinical Trial Registry (ChiCTR2100043260). Written consent was obtained from patients or their next of kin. The trial was registered prior to patient enrollment at the Chinese Clinical Trial Registry (ChiCTR2100043260; http://www.chictr.org.cn/showproj.aspx?proj=62662; Principal investigator: Keliang Xie; Date of registration: 9 February 2021; Date of Last Refreshed on: 28 May 2021).

### 2.2. Study Participants

The inclusion criteria were age ≥ 65 years; patients undergoing elective noncardiac, non-neurosurgical surgery; ASA (American Society of Anesthesiologists) I-III; duration of surgery ≤ 3 h; ethical, voluntary participation; and signed informed consent. The exclusion criteria were patients with a history of schizophrenia, epilepsy, Parkinsonism, or myasthenia gravis; pre-operative Simple State of Mind Questionnaire (SPMSQ) scores ≥ 8 errors or MMSE (Mini-Mental State Examination) scores less than 17; any cerebrovascular accidents within 3 months; severe cardiac and cerebrovascular disease; diabetic patients with diabetic complications; severe infections; SpO_2_ < 92% despite pre-operative oxygen at 5 L/min via nasal tube; participation in or exposure to the same neuropsychological scale within the last 30 days; or verbal communication impairment, hearing impairment, and inability to complete cognitive function tests.

### 2.3. Randomization and Masking

After giving consent, eligible patients were randomized using a web-based secure electronic central randomization system. Participants were randomized using minimization to either the hydrogen or sham (oxygen) group in a 1:1 allocation, and the results of randomization were saved and sealed until the study was finished. Trained outcome assessors and data collectors were blinded to group allocation throughout the study. Only the biostatistician and physician who administered the inhalation drugs knew the groups. A physician administered the inhaled drugs according to the randomization sequence. The device for inhaling hydrogen was hidden in a box. Trained outcome assessors, data collectors, and patients were blinded to group allocation throughout the study. If unexpected, rapid deterioration, or other emergencies happened (for example, hypoxia), we unmasked the group as necessary.

### 2.4. Procedures

General information about the patients was collected, including age, sex, weight, height, ASA classification, comorbidities, and medication status. For all patients, clear liquids (eg. water or electrolyte beverage) were prohibited for 2 h before surgery, and solid foods were prohibited for 8 h before surgery. The patients’ characteristics were recorded before surgery. Electrocardiogram (ECG), heart rate (HR), blood pressure (BP), oxygen saturation (SpO_2_), and bispectral index (BIS) were monitored in the operating room. All patients received general intravenous anesthesia, and routine laboratory tests were performed before surgery. SpO_2_ was routinely monitored after admission to the operating room, intravenous access was opened, and invasive arterial monitoring was connected after successful radial artery puncture. A physician who did not participate in the other aspects of the study administered the study drugs. In the control group, 33.3% oxygen was administered via a nasal cannula (0.5 L of pure oxygen mixed with 2.5 L of air) for 60 min at a flow rate of 3 L/min; in the hydrogen group, 66.7% hydrogen and 33.3% oxygen were administered via a nasal cannula using a hydrogen inhalation device and then inhaled for 1 h. Intraoperatively, the surgical method, operation time, duration of anesthesia, blood loss, urine volume, fluid volume, and other intraoperative conditions, such as hypertension, hypotension, tachycardia, bradycardia, and hypoxemia, were recorded for each patient. BIS was controlled at 40–60. Propofol and remifentanil infusion was stopped, and muscle relaxation antagonism was performed after the skin was sutured. Postoperative analgesia was provided by transvenous patient-controlled intravascular analgesia with sufentanil 0.03 µg/kg/h. Immediately after surgery, patients were admitted to the anesthesia resuscitation room. Given oxygen inhalation in the control group and hydrogen–oxygen mixture gas in the hydrogen group before anesthesia for 1 h. Adverse effects in the postanesthesia care unit (PACU), including hypoxemia, chills, nausea and vomiting (PONV), were recorded in both groups. If SPO_2_ was <90%, hydrogen inhalation was stopped and pure oxygen inhalation was given.

Additional analgesic medication (morphine or nonsteroidal anti-inflammatory drugs) was given for postoperative pain scores above 6. Nonpharmacological strategies were adopted for patients who developed postoperative delirium [16]. Haloperidol was used for patients with a severe agitation (RASS (Richmond Agitation-Sedation Scale) score of +3 or more) who were unresponsive to nonpharmacological treatment [17]. We prohibited the use of scopolamine and penehyclidine. Atropine was used only for reversing bradycardia.

### 2.5. Outcome Measures

During the postoperative period between discharge from the PACU to the seventh postoperative day, a diagnosis of one or more instances of delirium was defined as POD (postoperative delirium). Our primary outcome was the incidence of delirium within the first 7 days after surgery. The first assessment time point for postoperative delirium was 24 h after surgery to avoid diagnosing delirium that occurs after anesthesia [18,19]. We assessed delirium daily between 8:00–10:00 A.M. and 6:00–8:00 P.M. with the CAM (Confusion Assessment Method) until the seventh day after surgery. If the patient presented acute cognitive changes at other times, the team members were called by the nursing team, and the CAM was repeated.

Our secondary outcomes included length of stay in the hospital (from the first day after surgery to hospital discharge) and 30-day all-cause mortality. Additional outcomes were postoperative pain intensity, subjective sleep quality, and C-reactive protein (CRP). Pre- and postoperative pain was assessed using an NRS (numerical rating scale) score of 0–10 1 day before surgery and 1, 3, and 7 days after surgery. The quality of sleep was assessed 1 day before surgery and 1, 3, and 7 days after surgery at 8:00 A.M. using an NRS score of 0–10 [20,21]. An additional outcome was CRP. Laboratory tests, including routine blood, liver and renal function, electrolytes, and CRP, were repeated 1 day after surgery at 8 A.M.

Adverse events were recorded up until 24 h after surgery or until the end of the study. Bradycardia was defined as a heart rate less than 55 bpm or a decrease of more than 20% compared with the baseline. We defined hypotension as systolic blood pressure less than 90 mmHg or a decrease of more than 20% compared with the baseline. We defined hypertension as systolic blood pressure more than 160 mmHg or an increase of more than 20% compared with the baseline. Hypoxemia was defined as a pulse oxygen level less than 90%. Bradycardia and hypertension were addressed by using drug infusion, and drug infusion adjustments and fluid infusion were used to treat hypotension. Hypoxemia was addressed by an increased intake of oxygen and the use of a face mask.

### 2.6. Sample Size Calculation

In a previous study, the incidence of postoperative delirium was 28% [21]. In our preliminary experiment, the incidence of delirium was reduced by 40% when hydrogen was used for surgery patients. Thus, we considered that delirium could be reduced by 40% in the hydrogen group. Significance was set at 0.05, and power was set at 80% [22]. The loss-to-follow-up rate was 0.2, so we planned to enroll 184 patients.

### 2.7. Statistical Analysis

Categorical variables are presented as the number and percentage of patients. Continuous variables are presented as the mean and standard deviation (SD). A P-value of 0.05 was set as the threshold for statistical significance. Numeric variables were analyzed with an unpaired t-test. Categorical variables were analyzed using the χ^2^ test. Statistical analyses were performed with IBM^®^ SPSS^®^ Statistics Version 26 (IBM Deutschland GmbH, Ehningen, Germany) and GraphPad Prism 5.0 (GraphPad Software, Inc.; San Diego, CA, USA).

## 3. Results

**Figure 1:**
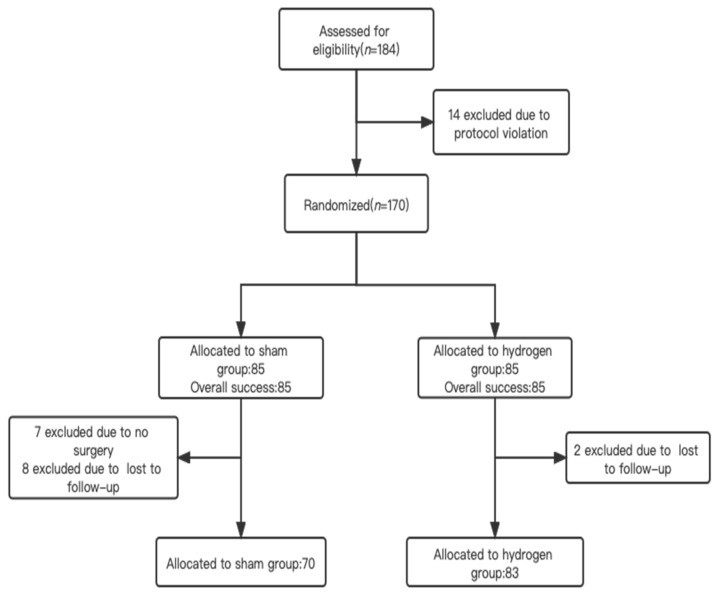
Patient recruitment.

From 10 January 2021 to 30 September 2021, a total of 170 patients meeting the inclusion criteria were enrolled in the study and randomly assigned to either the hydrogen (*n* = 85) or placebo (*n* = 85) group (Figure 1). There was no blinding lapse during this study. Ultimately, 153 patients completed the delirium assessment and were included in the final analyses (Figure 1). The final visit of the last randomized patient was on 20 September 2021.

Overall, the types of surgery for the 153 patients included orthopedic surgery, general surgery, and thoracic surgery. The baseline and pre-operative characteristics of the two groups were well matched with no significant differences in the patient demographics (Table 1). There were no significant differences in the length of surgery, intraoperative fluid volume, intraoperative urine volume, or intraoperative blood loss (Table 1).

**Figure 2:**
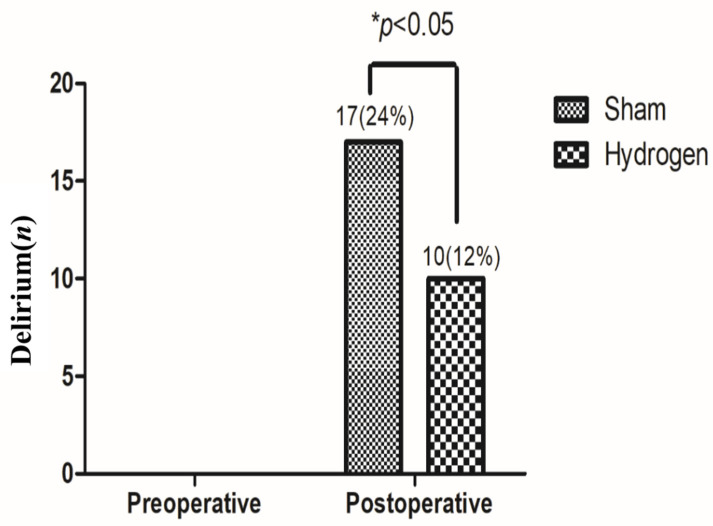
Number and percentage of diagnosed delirium pre-operatively and postoperatively in the two groups. For statistical significance, * indicates *p* ≤ 0.05.

Postoperative delirium happened in 17 (24%) of 70 patients who inhaled the placebo and in 10 (12%) of 83 patients who inhaled hydrogen. The incidence of delirium was decreased in the hydrogen group (Table 2, Figure 2). For most of these patients, delirium occurred almost 2 days after surgery and lasted for 1–2 days, but the duration of delirium and the onset of delirium did not differ between the groups (Table 3). Only five patients needed drug treatment during the 7-day postoperative period.

Regarding the secondary outcomes, no patient died for any reason in the two groups. No significant differences in the length of stay in the hospital after surgery were found between the two groups (Table 3).

**Figure 3:**
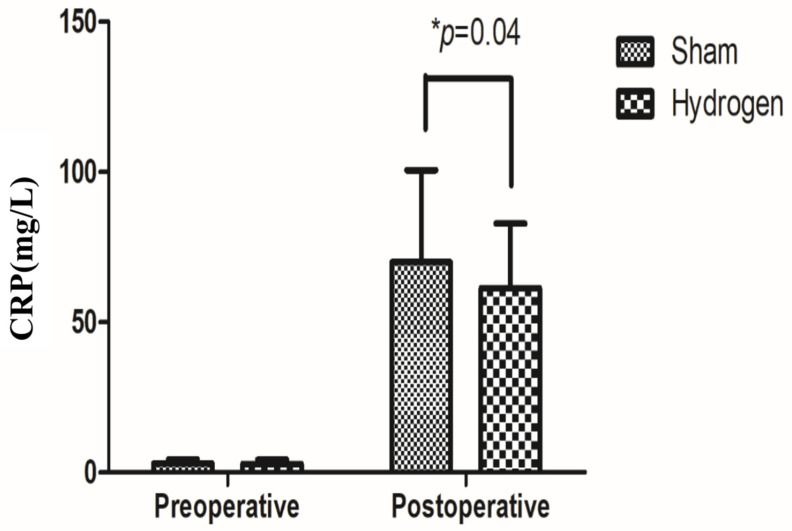
Pre- and postoperative CRP between the two groups. For statistical significance, * indicates *p* ≤ 0.05. CRP, C-reactive protein.

For the additional outcomes, there was no significant difference in sleep quality between the two groups at 1, 3, or 7 days postoperatively, and the sleep quality gradually recovered over 7 days postoperatively (Table 3). The NRS pain scores were higher in the hydrogen group (4.08 ± 1.77) than in the control group (3.54 ± 1.77) on day 1 (*p* < 0.05); however, the mean difference in NRS pain scores between the groups was small (1 to 1.6). There was no significant difference on day 3 or 7. The pain gradually decreased over 7 days (Table 3). The postoperative CRP level measured 1 day after surgery was significantly lower in the hydrogen group than in the control group (Figure 3).

The incidence of hypoxemia was higher in the hydrogen group (6[7%]) during hydrogen inhalation in the PACU than in the control group (2[3%]), and hypoxemia occurred in all patients undergoing thoracic surgery (Table 4). The incidence of the remaining adverse effects was essentially the same (Table 4).

## 4. Discussion

The pathophysiology of delirium is not fully understood, and we cannot explain the condition clearly using a single mechanism. Some studies emphasize the role of inflammation, particularly the effect of inflammatory factors on the blood–brain barrier and the effect of chronic stress on cytokines as well as cortisol. Other research [23] emphasizes imbalances in the central neurotransmitter transmission [23,24]. There is much research focusing on pharmacological agents for the prevention and treatment of delirium, but drugs inevitably have side effects. Hydrogen shows strong anti-oxidant activity and high tissue-transfer ability and can be safely applied to live animals and patients [25]. Therefore, in this study, prophylactic hydrogen inhalation was innovatively administered to elderly patients to determine whether it could reduce the occurrence of postoperative delirium.

Some studies have shown that delirium generally occurs within five days postoperatively [23]; this study showed that delirium occurred on average 2-3 days postoperatively, which is in line with previous findings. Most of the elderly people in this study had delirium lasting 1-2 days. The onset of delirium and the duration of delirium were similar in the hydrogen and control groups, so it is possible that hydrogen had little effect on the onset and duration of delirium. Additionally, we administered pharmacological interventions to elderly people who already had delirium but did not experience prolonged delirium, and there was no improvement. The occurrence of postoperative confusion was lower in the hydrogen group than in the control group, which may be related to reduced oxidative stress and pro-inflammatory cell production in the hydrogen group. It is known that postoperative pain can lead to delirium [26]; there was little difference in pain between patients at 1, 3, and 7 d postoperatively in this study. Sleep quality was worst at 1 d postoperatively and largely recovered by the 7th day, but it was also lower than pre-operative levels, possibly related to the hospital environment; however, there was no difference in sleep quality between the two groups. The disadvantage of postoperatively inhaled hydrogen is the potential for increased hypoxemia, especially in patients undergoing thoracic surgery; however, the hypoxemia was within manageable limits, and adjusting the oxygen concentrations could alleviate hypoxemia.

Some studies have shown that the incidence of delirium may be as high as 50% for major abdominal surgery, 53% for orthopedic surgery, and 12% and 13% for small- to medium-sized surgeries in ENT (ear, nose, and throat) and general surgery, respectively. Postoperative admission to the intensive care unit and mechanical ventilation are required in up to 80% of cases [27,28,29]. The incidence of delirium was 11% in the hydrogen group and 24% in the control group. The procedures we included were orthopedic and general surgery abdominal procedures, as well as thoracic surgery, with orthopedic surgery being the predominant type. In this study, hydrogen significantly reduced the incidence of delirium within 7 days postoperatively. Plasma CRP is a nonspecific marker of the acute-phase inflammatory response, infection, and tissue damage, all of which are associated with cognitive decline [29,30,31]. The association of delirium with inflammatory cytokines has been demonstrated and has been linked to the severity of delirium in relation to inflammation [32,33]. Studies have been performed to support the association of CRP with pre-operative and postoperative delirium [34]. Therefore, we assessed plasma CRP levels in two groups and found that elevated postoperative CRP levels were associated with POD and that hydrogen was able to reduce postoperative CRP levels. Many studies have shown that hydrogen is effective in the treatment of cerebrovascular disease and can reduce focal cerebral ischemia-reperfusion injury by reducing oxidative stress [33,35]. Hydrogen is able to increase survival in mice with cerebral embolism while reducing brain oedema [33,36]. It can also reduce neuronal damage and inhibit apoptosis in a cardiac arrest model [33,37]. In a model of subarachnoid hemorrhage, inhaled hydrogen improved neurological function by reducing oxidation and inflammation, reducing brain oedema, and disrupting the blood–brain barrier [38]. Many studies have shown that hydrogen also improves neurodegenerative lesions, which is also associated with its ability to prevent increases in oxidative stress and inflammatory factors [33,39,40].

Sleep deprivation and disturbed sleep are related to cognitive dysfunction. The incidence of sleep disturbances after major surgery is high. Sleep quality is not only related to postoperative pain but also to inflammatory mediators that may contribute to POCD [41]. For the Intensive Care Unit (ICU) patients, chronic anxiety can aggravate sleep disturbances. Improving the understanding of sleep and strengthening psychological support and environmental influence to reduce sleep disturbance can benefit the recovery of patients [42].

Delirium is also related to the experiences of patients and families. Families’ confidence in the recovery of a patient and the home environment help patients to recover from the delirium events [43]. This is also a factor worth paying attention to in the future.

## 5. Conclusions

Overall, the present study suggests that hydrogen can prevent postoperative delirium in elderly patients by reducing the inflammatory response of the body. The greatest advantage of hydrogen is that it is essentially free of side effects; however, as its mechanism remains unclear, further research is needed to investigate its role in preventing delirium.

## Figures and Tables

**Table 1:** Patient characteristics.

Patient Characteristic	Sham (*n* = 70)	Hydrogen (*n* = 83)	*p*_Value
Age (years) (mean ± SD)	70.74 ± 5.39	70.45 ± 5.20	0.73
Sex (%)			
Male	36(51%)	37(44%)	0.50
Female	34(49%)	46(56%)	
Weight (kg) (mean ± SD)	67.53 ± 11.86	66.94 ± 12.42	0.77
Height (cm) (mean ± SD)	165.49 ± 7.99	164.41 ± 8.12	0.41
ASA (%)			0.39
Ⅱ	37(53%)	37(44%)	
Ⅲ	33(47%)	46(56%)	
Hgb	127.56 ± 14.47	127.81 ± 16.07	0.92
PLT	215.5 ± 62.27	230.06 ± 51.72	0.12
ALB	36.87 ± 4.10	37.66 ± 3.80	0.22
ALT	19.79 ± 16.22	18.01 ± 10.02	0.41
AST	20.09 ± 9.45	18.67 ± 13.20	0.28
GLU	5.62 ± 1.35	5.45 ± 1.51	0.48
Hypertension	12(17%)	10(12%)	0.37
Diabetes	12(17%)	10(12%)	0.37

Pre-operative patient demographics. Dichotomous data are presented as the number and percentage of patients. Continuous data are presented as the mean and standard deviation. SD, standard deviation. Hgb: Hemoglobin; PLT: Platelet; ALB: Albumin; ALT: Alanine transaminase; AST: Aspartate transaminase; GLU: Glucose.

**Table 2:** Perioperative variables.

	Sham (*n* = 70)	Hydrogen (*n* = 83)	*p*_Value
Type of surgery			
orthopedic	50(71%)	53(63%)	
abdominal	15(21%)	18(22%)	
thoracic	5(8%)	12(15%)	
Duration of surgery (min) (mean ± SD)	117.77 ± 84.48	116.08 ± 50.51	0.88
Total intra-operative infusion (ml) (mean ± SD)	1391.43 ± 416.85	1389.16 ± 434.49	0.20
Urine volume (ml) (mean ± SD)	308.57 ± 256.50	348.57 ± 287.97	0.37
Bleeding volume (ml) (mean ± SD)	154.14 ± 327.50	83.72 ± 114.43	0.07
Use of other analgesic during the first 7 postoperative days			
Morphine (*n*)	1(1%)	1(1%)	0.90
Morphine (mg)	10	10	
Flurbiprofen axetil (*n*)	24(34%)	18(21%)	0.08
Flurbiprofen axetil (mg)	104.17 ± 44.03	100 ± 34.3	0.74

Dichotomous data are presented as the number and percentage of patients. Continuous data are presented as the mean and standard deviation. SD, standard deviation.

**Table 3:** Effectiveness outcomes.

	Sham (*n* = 70)	Hydrogen (*n* = 83)	*p*_Value
Primary endpoint			
Overall incidence of delirium	17(24%)(95% CI = 1.49 to 1.67)	10(12%)(95% CI = 1.18 to 1.57)	0.048 *
Secondary endpoints			
Length of stay in the hospital after surgery (day)	9.70 ± 2.18	9.23 ± 1.90	0.16
All-cause 30-day mortality	0(0%)	0(0%)	
Prespecified analyses			
NRS for pain (score)			
First morning after surgery	3.54 ± 1.77	4.08 ± 1.77	0.048 *
Third morning after surgery	2.15 ± 1.11	2.2 ± 1.10	0.82
Seventh morning after surgery	1.06 ± 0.99	1.08 ± 0.91	0.84
NRS for sleep quality (score)			
First morning after surgery	5.21 ± 1.84	5.24 ± 1.64	0.93
Third morning after surgery	6.11 ± 1.50	5.95 ± 1.53	0.50
Seventh morning after surgery	6.50 ± 1.43	6.41 ± 1.53	0.71
Exploratory analyses			
Time to onset of delirium (day)	2.29 ± 1.26	2.45 ± 1.21	0.74
Duration of delirium (day)	1.71 ± 0.69	1.36 ± 0.67	0.21
Haloperidol treatment	2(2.8%)	3(3.6%)	

Continuous data are presented as the mean and standard. For statistical significance, * indicates *p* ≤ 0.05.

**Table 4:** Postoperative adverse effect data.

	Sham (*n* = 70)	Hydrogen (*n* = 83)	*p*_Value
SPO_2__PACU < 92% (*n*)(%)	2(3%)	6(7%)	0.23
PONV (*n*)(%)	5(7%)	4(5%)	0.55
Headache (*n*)(%)	0(0%)	0(0%)	
Uroschesis (*n*)(%)	0(0%)	0(0%)	
Bradycardia (*n*)(%)	0(0%)	1(1%)	0.36
Hypotension (*n*)(%)	2(3%)	3(4%)	0.80
Hypertension (*n*)(%)	2(3%)	3(4%)	0.80

Number and percentage of postoperative adverse effects. PONV, postoperative nausea and vomiting.

## Data Availability

The datasets generated and/or analysed during the current study are available in the [Chinese Clinical Trial Registry] repository, [ChiCTR2100043260, URL: http://www.chictr.org.cn/edit.aspx?pid=62662&htm=4; Principal investigator: Keliang Xie; Date of registration: 9 February 2021; Date of Last Refreshed on: 28 May 2021]. The datasets used and/or analysed during the current study available from the corresponding author on reasonable request.

## Abbreviations

POD, postoperative delirium; PACU, postanesthesia care unit; PONV, postoperative nausea and vomiting; CAM, the Confusion Assessment Method; CRP, C-reactive protein.
